# Likelihood of Participation in Home-Based Cognitive Assessment: The Role of Subjective Cognitive Decline and Age

**DOI:** 10.1093/geroni/igaa057.940

**Published:** 2020-12-16

**Authors:** Moriah Splonskowski, Holly Cooke, Claudia Jacova

**Affiliations:** 1 Pacific University, Forest Grove, Oregon, United States; 2 Pacific University, Hillsboro, Oregon, United States

## Abstract

Home-based cognitive assessment (HBCA) services are emerging as a convenient alternative to in-clinic cognitive assessment and may aid in mitigating barriers to detecting cognitive impairment (CI). It is unknown which older adults would be likely to participate in HBCA. Here we investigated the role of age and Subjective Cognitive Decline (SCD). SCD has demonstrated an increased risk for progression to CI/dementia. A nation-wide community-dwelling sample of 494 adults age 50+ were recruited via Amazon Mechanical Turk to complete an online survey assessing perceptions around HBCA and SCD. Our sample was 91.9% White and 66.8% female. It consisted of 174 respondents aged 50-60, 265 aged 61- 70, and 55 aged 71-79. Age groups were comparable with respect to their acceptance of cognitive assessment (Range 4-20, higher score=higher acceptance, 7.9±3.3, 8.15±3.2, 8.05±3.43) and SCD-Q total (43.1±5.8, 43.2±5.7, 43.3±5.7). Correlation analysis revealed a relationship between SCD-QSCD total and perceived likelihood of participation in HBCA for those ages 61-70 (r(263) = .222 p = .000), but not for ages 50-60 or 71-79 (r(172) = .102 p = .152; r(53) = -.102 p = .458). Our findings suggest that SCD influences the likelihood of participation in HBCA for older adults’ transitioning to old age (61-70). Findings show that for adults transitioning into old age (61-70), perceived cognitive state influences their likelihood of participation in HBCA. Importantly, concerns about CI/dementia may generate more favorable perceptions of HBCA for this group.

